# The diabetes health plan and medication adherence among individuals with low incomes

**DOI:** 10.1111/1475-6773.13992

**Published:** 2022-05-02

**Authors:** Kimberly Danae Cauley Narain, Norman Turk, O. Kenrik Duru, Tannaz Moin, Sam Ho, Carol M. Mangione

**Affiliations:** ^1^ Division of General Internal Medicine and Health Services Research, Department of Medicine, David Geffen School of Medicine University of California Los Angeles California USA; ^2^ Center for Health Advancement Fielding School of Public Health, University of California Los Angeles Los Angeles California USA; ^3^ Center for the Study of Racism Social Justice, and Health Los Angeles Los Angeles California USA; ^4^ HSR&D Center for the Study of Healthcare Innovation, Implementation & Policy VA Greater Los Angeles Healthcare System Los Angeles California USA; ^5^ UnitedHealthcare Minnetonka Minnesota USA; ^6^ Fielding School of Public Health University of California Los Angeles California USA

**Keywords:** diabetes, health disparities, health equity, low‐income, medication non‐adherence, value based insurance design

## Abstract

**Objective:**

To test the impact of the Diabetes Health Plan (DHP), a diabetes‐specific insurance plan that lowers out‐of‐pocket costs for diabetes‐related medications and clinical visits, on adherence to oral hypoglycemic medications among low‐income adults with Type 2 Diabetes (T2DM).

**Data Sources and Study Setting:**

Cohort of adults (18–64) with T2DM, an annual household income <USD 30,000, and who were continuously enrolled in an employer‐sponsored UnitedHealthcare plan for at least two years between 2009 and 2014.

**Study Design:**

We employed a linear regression Difference‐In‐Differences (DID) approach with a matched comparison group. To assess for differential DHP effects across adherent versus non‐adherent patients, we ran a Difference‐in‐Difference‐in‐Differences (DDD) analysis by including an interaction term that included indicators for DHP exposure status and time, and low versus high baseline medication adherence.

**Data Collection:**

The analytic data set is limited to employer groups that purchased the DHP and standard benefit plans from UnitedHealthcare, had internal pharmacy contracts; complete pharmacy claims data, and sufficient medical claims and lab data to identify employees and their dependents with T2DM.

**Principal Findings:**

Our DID analysis did not show improved medication adherence associated with employer DHP adoption. However, the DDD model suggested a difference between DHP‐exposed and comparison beneficiaries when comparing the relative effect on individuals who were adherent versus non‐adherent at baseline, as suggested by the significant three‐way interaction term (10.2,*p* = 0.028). This effect was driven by the 8.2 percentage point increase in medication adherence for the DHP subsample that was non‐adherent at baseline.

**Conclusions:**

The DHP may benefit low‐income patients with low baseline medication adherence. Value‐based insurance design may be an important strategy for mitigating income disparities in T2DM outcomes.

**What is known on the topic**

- Individuals with low incomes suffer disproportionate diabetes‐related morbidity and mortality.
- Cost‐related medication non‐adherence is an important driver of the income gradient in diabetes‐related health outcomes.
- Value‐based insurance design (VBID) strategies have shown promise for mitigating income‐based disparities in several clinical contexts.

**What this study adds**

- This is the first study to show an improvement in hypoglycemic medication adherence associated with a diabetes‐specific insurance plan, among individuals with low household income and low baseline medication adherence.

## 1 INTRODUCTION

While the prevalence of diabetes continues to increase in the United States, there is a steep socioeconomic gradient in diabetes‐related morbidity and mortality.^1^ Having a family income below the Federal Poverty Level (FPL) is associated with a two‐fold increase in an individual's risk of diabetes‐related mortality, relative to an individual with a household income ≥400% FPL.^2^ Medication non‐adherence due to costs is an important driver of this socioeconomic gradient in diabetes outcomes. Although isolated copayments for medications and medical visits may be low, when considered in the aggregate these costs may pose a financial burden for individuals with low incomes, forcing tradeoffs between medical care and basic necessities.^3^ The positive relationship between low oral hypoglycemic medication adherence among diabetes patients, hospitalization, and emergency room utilization is well‐documented.^4^

In 2009, UnitedHealthcare (UHC) introduced the first Diabetes Health Plan (DHP), a condition‐specific plan based on principles of value‐based health insurance benefit design. The DHP includes financial incentives to encourage patient engagement in evidence‐based diabetes care, including reduced or eliminated out‐of‐pocket patient expenses for diabetes‐related physician visits; free diabetes self‐monitoring training and supplies; and reduced or eliminated out‐of‐pocket expenses for diabetes‐related medicines.^5^, ^6^ The DHP also provides access to diabetes‐specific telephone case management as well as other online resources. Additionally, the DHP provides scorecards with reminders to complete health maintenance activities, such as biannual hemoglobin A1C and cholesterol screening, and an annual retinal eye exam. Overall, the DHP provides between USD 150 and 500 in annual out‐of‐pocket savings for enrollees.^7^

The DHP standard benefit design can be modified by purchasing employers to better suit the needs of beneficiaries, which includes both employees and their dependents. Some employers use an opt‐in enrollment strategy while others use an opt‐out strategy. Studies have shown that DHP uptake can range from a low of 8% among opt‐in plans to a high of 85% for opt‐out plans.^8^ Additionally, studies have shown variability in the demographic characteristics of DHP participants as a consequence of the enrollment strategy. Specifically, Kimbro et al. found that DHP participants enrolled in an opt‐out plan were more likely to be dependents, were more racially and ethnically diverse, and had a broader range of incomes and educational backgrounds relative to participants enrolled in opt‐in plans, who tended to have higher incomes, more education and who were less likely to be Hispanic.^6^

To our knowledge, only one study has examined the effects of the DHP on medication adherence. Duru et al. conducted an inverse propensity score‐weighted Difference‐In‐Differences (DID) study that exploited variability in employer purchase of the DHP, to examine the impact of the DHP on employer‐level medication adherence and found that DHP purchase was associated with a 4‐percentage point increase in medication adherence, after one year.^8^ However, the effects of the DHP across income levels have not been investigated. Individuals with low incomes may be more sensitive to out‐of‐pocket costs, resulting in stronger effects of the DHP among this population.^9^ Additionally, studies have shown that the magnitude of the effect of value‐based insurance design initiatives on medication adherence is largest among individuals with the lowest baseline medication adherence.^10^ Furthermore, individuals with the lowest medication adherence are at the greatest risk for diabetes‐related morbidity and mortality.^4^

To address these evidence gaps, we conducted a DID study with a propensity‐matched comparison group among individuals with annual household incomes ≤30,000, which takes advantage of the variability in DHP purchases across employers. A household income of 30,000 falls below the FPL threshold for a family of 6.^11^ Additionally, we used a Difference‐in‐Difference‐in‐Differences (DDD) study design to assess for differential DHP effects across baseline medication adherence levels.

## 2 METHODS

### 2.1 Data source and study population

The analytic data set is limited to 26 large employer groups that purchased the DHP and standard benefit plans from UHC (2009–2014) that have (1) internal pharmacy contracts, (2) complete pharmacy claims data, (3) sufficient medical claims and lab data to identify employees with Type 2 diabetes (T2DM) and (4) fewer than 15% of employees enrolled in High Deductible Health Plans. In addition to the above‐mentioned criteria, the comparison employer groups are further limited to those that have overlapping propensity scores with DHP employers after employer‐level matching (described further below) and who have at least two years of continuous enrollment in the standard benefit plan during the duration of the match to the DHP employer.

**FIGURE 1:**
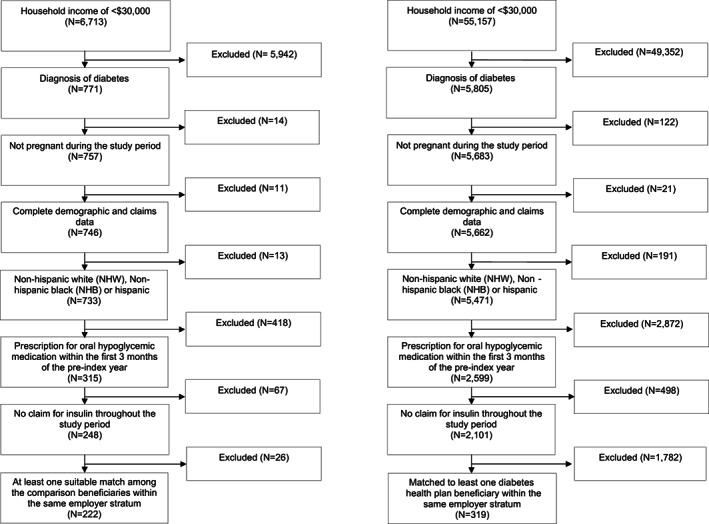
Sample size flow chart

A diabetes diagnosis was defined as having any of the following prior to the implementation of the DHP: (1) at least one 250 X ICD‐9 diagnosis code from an inpatient, outpatient, or emergency department claim; (2) hemoglobin A1C laboratory value of 6.5% or greater or a 2‐h value on an oral glucose tolerance test of greater than 200 mg/dL, or (3) at least one prescription fill for an oral hypoglycemic medication other than metformin. Estimated household income is obtained from the AmeriLINK data.^12^ Income data are collected by a monthly survey from a representative cross‐section of the US population of >30,000 households and are informed by 130 variables that encompass ZIP +4 (a highly specific geographic locator), Internal Revenue Service data, address‐level home value, aggregated credit, and short‐term loans. Derived estimates of household income are validated by comparison to self‐reported income collected by household surveys.^13^ Our final sample includes 222 DHP and 280 unique comparison beneficiaries for a total of 319 matches with replacement (Figure 1).

### 2.2 Propensity score matching

Matching criteria for both the DHP and comparison employers were derived with respect to the 12‐month period preceding the date of DHP adoption for the DHP employers (the index date). The matching criteria included the following as reported by UHC: average employee salary, geographic region, number of employees, percentage female, percentage in each racial/ethnic category (White, Black, Asian, and Hispanic), health benefit plan generosity, percentage of employees with an high deductible health plan (HDHP), percentage of beneficiaries with each one of the following claims‐based co‐morbidities (hypertension, hyperlipidemia, coronary artery disease, anxiety/depression, dementia, osteoarthritis, rheumatoid arthritis, non‐skin cancer, chronic obstructive pulmonary disease, congestive heart failure, atrial fibrillation, end‐stage renal disease, peripheral vascular disease, stroke, schizophrenia). The resultant scores of the employer‐level propensity match that were within the region of common support were divided into 5 strata. Matched control employees were pulled from control employers that shared the same stratum of the DHP employer for whom the DHP employee was being matched. A single comparison employer could be matched to more than one DHP employer. Individual‐level matching criteria were based on the following pre‐index date criteria: race and ethnicity, age, gender, Charlson Comorbidity Index, presence of any diabetes complication (retinopathy, nephropathy, neuropathy, cardio/peripheral vascular disease, history of a diabetes‐related hospitalization) and baseline adherence to oral hypoglycemic medications. Nearest neighbor matching was conducted with replacement using a caliper equal to 25% of the propensity score standard deviation, in an effort to get 3 comparison matches for each DHP beneficiary.^14^ The employer and beneficiary matching was done using PROC PSMATCH in SAS version 9.4.

### 2.3 Outcomes

Medication adherence was calculated as the mean proportion of days covered (PDC) over the last 9 months of the post‐period, accounting for medication carry‐forward from the first quarter. We did not control for fills for multiple prescriptions within a drug class, although if two or more prescriptions were filled on the same day, we included only the prescription with the higher days' supply in the adherence calculation. This was the same approach taken by Duru et al. to calculate this measure with the exception that it is measured at the individual rather than the employer‐level.^8^

### 2.4 Statistical analysis

We used a DID study to examine the impact of the DHP on oral hypoglycemic medication adherence. The key assumption of the DID study is the parallel trends assumption which necessitates that the pre‐intervention trends for outcome measures across the treatment and comparison groups are the same.^16^ If the parallel trend assumption is met, any difference in the pre‐post intervention change in slope across treatment and comparison groups is attributed to intervention effects. We use the propensity‐matched sample to increase the likelihood that the DHP and comparison groups have a similar trend of medication adherence during the pre‐intervention time period. The statistical model is an ordinary least squares regression (OLS) model with standard errors adjusted for clustering of observations within individuals, using generalized estimating equations (GEE).^15^ This modeling approach yields estimates of the average treatment effect on the treated (ATT) of the DHP.

The DID model takes the following form:

$${\text{Adherence}}_{\mathrm{it}}=\beta_{0\mathrm{it}}+\beta_{1}\left({\mathrm{DHP}}_{i}\right)+\beta_{2}\left({\text{Post}}_{t}\right)+\beta_{3}\left({\mathrm{DHP}}_{i}\right)\left({\text{Post}}_{t}\right)+\varepsilon_{\mathrm{it}}.$$

where ${\text{Adherence}}_{\mathrm{it}}$ (adherence of person *i* at time period *t*) is the oral hypoglycemic medication adherence as measured using the PDC, $\beta_{0\mathrm{it}}$ is the average adherence in the comparison group during the pre‐index period, $\beta_{1}$ is the difference in adherence between the DHP and the comparison group during the pre‐index period, $\beta_{2}$ is the average change in adherence in the comparison group across the pre and post‐index periods. $\beta_{3}$, the estimate of interest, is the difference in the pre/post‐index date slope changes in medication adherence across the DHP and comparison groups. We interpret a positive and statistically significant $\beta_{3}$ as a positive impact of the DHP on medication adherence. To assess for differential DHP effects across baseline medication adherence, we then incorporate an interaction term that includes indicators for DHP exposure status, time, and low (PDC ˂80%) versus high baseline medication adherence (PDC ≥80%) into the above‐mentioned models.

The DDD model takes the following form:

$${\text{Adherence}}_{\mathrm{it}}=\beta_{0\mathrm{it}}+\beta_{1}\left({\mathrm{DHP}}_{i}\right)+\beta_{2}\left({\text{Post}}_{t}\right)+\beta_{3}\left({\mathrm{DHP}}_{i}\right)\left({\text{Post}}_{t}\right)+\beta_{4}\left({\mathrm{NAD}}_{i}\right)+\beta_{5}\left({\mathrm{NAD}}_{i}\right)\left({\mathrm{DHP}}_{i}\right)+\beta_{6}\left({\mathrm{NAD}}_{i}\right)\left({\text{Post}}_{t}\right)+\beta_{7}\left({\mathrm{NAD}}_{i}\right)\left({\mathrm{DHP}}_{i}\right)\left({\text{Post}}_{t}\right)+\varepsilon_{\mathrm{it}}.$$

where ${\text{Adherence}}_{\mathrm{it}}$ is defined per above, $\beta_{0\mathrm{it}}$ is the average medication adherence in the comparison group, among individuals classified as adherent, during the pre‐index period, $\beta_{1}$ is the average difference in adherence between the DHP and the comparison group during the pre‐period, among beneficiaries classified as adherent, $\beta_{2}$ is the average change in adherence in the comparison group across the pre and post‐index periods, among beneficiaries classified as adherent. $\beta_{3}$, is the average difference in the pre/post‐index date changes in medication adherence, across the DHP and comparison group, among beneficiaries classified as adherent. $\beta_{4}$ is the average medication adherence in the comparison group, among beneficiaries who are classified as non‐adherent, during the pre‐index period, $\beta_{5}$ is the average difference in adherence between the DHP and the comparison group during the pre‐period, among beneficiaries classified as non‐adherent, $\beta_{6}$ is the average change in adherence in the comparison group, across the pre and post‐index periods among beneficiaries classified as non‐adherent. $\beta_{7}$, the coefficient of interest, is the average difference in the pre/post‐index date changes in medication adherence across the DHP and comparison beneficiaries, classified as non‐adherent. If the coefficient for $\beta_{7}$ is positive and statistically significant there will be support for our hypothesis of stronger DHP effects among beneficiaries with lower baseline medication adherence. Lastly, we conducted an additional test to assess the sensitivity of our results to selection bias by repeating the above‐mentioned analyses with DHP employers that use an opt‐out enrollment strategy as the sole source of the treatment population. This methodological change should allow for evaluation of the DHP medication adherence effects among a less motivated subset of beneficiaries than the subset mostly comprised of individuals that proactively enrolled in the DHP.^6^ All data was anonymized and therefore this study was not subject to Institutional Board Review.

### 2.5 Results

The final analytic sample included 319 matches with replacement (Table 1). In the overall unmatched sample, DHP beneficiaries were slightly older (54.9 vs. 53.8 years) and a greater percentage of DHP beneficiaries were female (58.6% vs. 49.4%) versus comparison beneficiaries. Additionally, a greater proportion of DHP beneficiaries were Non‐Hispanic Black (NHB) (41.0% vs. 22.6%) and a smaller proportion was Hispanic (13.1% vs. 32.2%) versus comparison beneficiaries. In the unmatched sample with low baseline adherence (Table 2), racial and ethnic differences persisted across the DHP and comparison beneficiaries, with a similar pattern to what was observed among the total sample. The DHP beneficiaries were more likely to have any diabetes complications (40.7% vs. 30.0%) and had lower baseline medication adherence (48.7% vs. 53.3%). After propensity score matching, there were no statistically significant differences between DHP exposed beneficiaries and comparison beneficiaries in demographic characteristics. Post‐matching means standardized differences for all covariates included in the propensity score models are <0.1, across the DHP and comparison beneficiaries, indicating sufficient matching.^14^

**TABLE 1:** Descriptive statistics by treatment status for total population (unmatched and matched)

Covariates	Unmatched comparison sample (*N* = 1347)	*p*‐value	DHP population (*N* = 222)	Matched comparison sample (*N* = 319)	*p*‐value
Mean age (SD)	53.8 (8.0)	0.044	54.9 (7.5)	54.8 (5.9)	0.777
Female	49.4%	0.012	58.6%	57.1%	0.761
Race/ethnicity					
Hispanic	32.2%	<0.001	13.1%	15.3%	0.747
Black	22.6%		41.0%	41.4%	
White	45.2%		45.9%	43.2%	
Any diabetes complication (yes/no)	33.8%	0.026	41.4%	43.6%	0.643
Charlson co‐morbidity index (SD)	1.6 (1.2)	0.114	1.7 (1.5)	1.7 (1.1)	0.508
Unadjusted adherence (pre)	78.3 (25)	0.347	76.6 (26)	78.1 (21)	0.521

*Note*: Propensity scores were generated using logistic regression models that included the following pre‐index date criteria: race/ethnicity, age, gender Charlson co‐morbidity index, diabetes complication index (retinopathy, nephropathy, neuropathy, cardio/peripheral vascular disease, history of a diabetes‐related hospitalization), and baseline medication adherence to oral hypoglycemic medications. Nearest neighbor‐matching was conducted with replacement using a caliper of 25% SD of the propensity score in an effort to get 3 comparison matches for each DHP employee. Bivariates were generated using a t‐test and Chi‐squared test for continuous and categorical/dichotomous variables, respectively.

Abbreviation: DHP, diabetes health plan.

**TABLE 2:** Descriptive statistics by treatment status for the population with low baseline medication adherence (unmatched and matched)

Covariates	Unmatched comparison sample (*N* = 546)	*p*‐value	DHP population (*N* = 86)	Matched comparison sample (*N* = 122)	*p*‐value
Mean Age (SD)	51.3 (8.7)	0.072	53.1 (7.8)	52.1 (6.7)	0.347
Female	54.4%	0.293	60.5%	67.3%	0.344
Race/ethnicity					
Hispanic	37.7%	<0.001	15.1%	14.8%	0.898
Black	24.4%		47.7%	44.7%	
White	37.9%		37.2%	40.5%	
Any diabetes complication (yes/no)	30.0%	0.048	40.7%	37.2%	0.637
Charlson co‐morbidity index (SD) (SD)	1.5 (1.3)	0.256	1.7 (1.6)	1.5 (0.8)	0.211
Unadjusted adherence (pre)	53.3 (20)	0.043	48.7 (19)	51.8 (17)	0.230

*Note*: Low adherence is defined as a proportion of days covered (PDC) < 80% over the last 9 months. Propensity scores were generated using logistic regression models that included the following pre‐index date criteria: race/ethnicity, age, gender Charlson co‐morbidity index, Diabetes complication index (retinopathy, nephropathy, neuropathy, cardio/peripheral vascular disease, history of a diabetes‐related hospitalization), and baseline medication adherence to oral hypoglycemic medications. Nearest neighbor‐matching was conducted with replacement using a caliper of 25% SD of the propensity score in an effort to get 3 comparison matches for each DHP employee. Bivariates were generated using a t‐test and Chi‐squared test for continuous and categorical/dichotomous variables, respectively.

Abbreviation: DHP, diabetes health plan.

In adjusted results of the model that did not include baseline medication adherence (Table 3), the changes in mean predicted adherence rates over time among DHP beneficiaries were similar to those among comparison beneficiaries. Specifically, the *p*‐value of the DID estimator (the coefficient for the interaction between the DHP status and time indicators) was not statistically significant. However, the DDD model (Table 4) suggested a difference between DHP beneficiaries and comparison beneficiaries when comparing the relative effect of baseline adherence, as suggested by the statistically significant three‐way interaction term (*p*‐value = 0.028). This effect is primarily driven by the projected 8.2% point increase in medication adherence among DHP‐exposed beneficiaries with low baseline medication adherence. To facilitate a more direct interpretation of the effect of the DHP among beneficiaries with low household incomes and low baseline medication adherence, we estimated an additional DID model among this population. Consistent with the above‐mentioned results, DHP‐exposure was associated with an 8.2%‐point increase in medication adherence (*p*‐value = 0.042, results not shown).

**TABLE 3:** Predicted change in oral hypoglycemic adherence with DHP exposure, relative to no exposure, (difference‐in‐differences)

Enrollment strategy	Opt‐In^a^	Opt‐Out^b^
DHP	−0.6 (−4.1 to 2.8)	−3.7 (−7.9 to 0.4)
Comparison	−2.3 (−5.0 to 0.4)	−1.7 (−4.3 to 0.9)
Absolute difference	+1.7 (−2.7 to 6.0)	−2.1 (−7.0 to 2.8)
*p*‐value	0.451	0.393

*Note*: The point estimates reflect percentage point changes in the predicted adherence measure.

Abbreviation: DHP, diabetes health plan.

^a^ DHP *N* = 222; Control *N* = 319.

^b^ DHP *N* = 185; Control *N* = 263.

**TABLE 4:** Predicted change in oral hypoglycemic adherence with DHP exposure, relative to no exposure, by baseline adherence (difference‐in‐difference‐in‐differences)

Enrollment Strategy	Opt‐In		Opt‐Out	
Low adherence (PDC < 80%)	DHP *N* = 86 Ctrl *N* = 122	+8.2 (0.3 to 16.0)	DHP *N* = 69 Ctrl *N* = 107	+7.0 (−2.1 to 16.0)
High adherence (PDC ≥ 80%)	DHP *N* = 136 Ctrl *N* = 197	−2.0 (−6.5 to 2.5)	DHP *N* = 116 Ctrl *N* = 156	−6.6 (−11.7 to −1.4)
Absolute difference		+10.2 (1.1 to 19.0)		+13.6 (3.1 to 24.0)
*p*‐value		0.028		0.011

*Note*: The point estimates reflect percentage point changes in the predicted adherence measure.

Abbreviation: Ctrl, matched control; DHP, diabetes health plan; PDC, proportion of days covered.

In sensitivity analyses including only employers enrolling DHP beneficiaries using an opt‐out strategy as the treatment population, once again, we find that there is no association between DHP‐exposure and medication adherence among the broader population of beneficiaries. However, the DDD model assessing the effects of DHP‐exposure with respect to baseline medication adherence once again shows a statistically significant positive association between DHP exposure and medication adherence among the subset of the population with low baseline medication adherence (Table 4). We also opted to test the sensitivity of the DDD results to our ≤30 K income threshold by repeating the analysis using a ≤40 K income threshold. The results of this model suggested a small insignificant trend in favor of the DHP (Table S1).

## 3 DISCUSSION

Using strong quasi‐experimental study designs, we evaluated the relationship between the DHP exposure and medication adherence among low‐income beneficiaries, including with respect to baseline medication adherence. Our finding of a positive relationship between DHP exposure and oral hypoglycemic medication adherence in the low‐income subpopulation with low baseline medication adherence makes an important contribution to the literature, as it is the first individual‐level study, to our knowledge, to show the beneficial effects of DHP exposure on medication adherence. Additionally, the benefit of the DHP is observed among a subpopulation that is at extremely high risk for diabetes‐related morbidity and mortality.^2^ The robustness of the results to sensitivity analyses conducted only using DHP beneficiaries enrolled using an opt‐out strategy is also reassuring because it increases confidence that the beneficial medication adherence effects observed among the DHP‐exposed beneficiaries do not merely reflect an artifact of selection bias.

The study results must be viewed in the context of some important limitations. The relationship between the DHP and medication adherence is observed across a relatively short time frame and consequently, this relationship may change over time. Our medication adherence outcome reflects prescription fills rather than actual ingestion of medications and therefore may not be as valid as pill counting or the use of a smart pill bottle for measuring medication adherence. The validity of our findings would be bolstered by DHP‐associated improvements in HbA1c control; however, our access to lab results is incomplete. Given that we do have access to all the claims for the HbA1c test, we instead conduct an additional DDD analysis of the relationship between DHP exposure and HbA1c testing frequency (Table S2). The results of this analysis suggest a non‐significant trend toward a decline in HbA1c testing frequency among the DHP exposed cohort, relative to the control cohort. One potential explanation for this finding is that the reduced testing trend reflects better HbA1c control in the context of improved medication adherence. Guidelines suggest that HbA1c testing frequency can be reduced to every 6 months among individuals meeting HbA1c goals, otherwise it should be checked every three months.^17^ However, without having access to all of the lab data, it is not possible to definitively prove this theory. Additionally, a key underlying assumption of the DID/DDD approach is that secular time trends for the treatment and comparison group do not differ in the pre‐treatment time period. We use propensity score matching in an effort to ensure similarity of pre‐treatment secular time trends across the DHP‐exposed and comparison beneficiaries but this strategy does not account for differences in unmeasurable factors. Our study approach also reflects an intent‐to‐treat design which may result in beneficiaries without DHP insurance coverage being included in the treatment population. Consequently, DHP effect estimates may be biased towards the null. However, this intent‐to‐treat design reduces the risk of selection bias being a plausible explanation for our results. Lastly, our results reflect average effect estimates for the DHP. As such, effects may vary across DHP implementation strategies.

Despite the above‐mentioned limitations, this study may have some important implications for population health and health equity. The magnitude of the estimated effect of DHP on medication adherence is clinically meaningful. The projected 8.2%‐point increase in medication adherence among the subpopulation with low baseline adherence represents a nearly 17% increase in adherence above baseline (mean PDC = 47.7%). Studies suggest that a 10% increase in medication adherence is associated with a 0.14–0.16 decrease in HbA1c.^18^, ^19^ Shenolikar et al. found that a 10% increase in oral hypoglycemic medication adherence was associated with a 6.9% decrease in the risk of hospitalization and a 5.1% decrease in the risk of an emergency room visit.^20^ As such, incorporating VBID principles into insurance benefit design may be a useful strategy to help reduce the burden of diabetes‐related morbidity and mortality among the most heavily affected population. Additionally, it is worth reiterating that lower out‐of‐pocket costs are not the only feature of the DHP. Other aspects of the DHP designed to promote patient engagement such as access to diabetes‐specific telephone case management as well as the provision of scorecards and health maintenance reminders may be facilitating medication adherence by improving health literacy, self‐management knowledge, and self‐efficacy.^21^ While we do not address the cost of DHP implementation in this study, this may be a particular area of concern for employers considering insurance plans such as the DHP. Ideally, the cost of DHP implementation could be lowered by targeting medication copayment subsidies to low‐income beneficiaries with low medication adherence; however, matching copayments to changing adherence levels would likely prove too administratively complex to be useful. Additionally, this strategy may create perverse incentives to reduce medication compliance among beneficiaries. Another consideration to reduce the cost of the DHP is the use of sliding scale copayments based on income. Examples of this strategy in the employer‐sponsored insurance context are limited but this is a strategy that was implemented during healthcare reform in Massachusetts and that is prevalent throughout provinces in Canada.^22^, ^23^ At the same time, more DHP advertising and administrative support could be diverted away from higher‐income beneficiaries and toward lower‐income beneficiaries.

## 4 CONCLUSION

We used strong quasi‐experimental studies and administrative/pharmacy claims data to evaluate the effect of the Diabetes Health Plan (the first disease‐specific health plan based on value‐based health insurance benefit principles) on oral hypoglycemic medication adherence among beneficiaries with household income ≤30,000 and found a clinically meaningful improvement in medication adherence among DHP‐exposed beneficiaries with low baseline medication adherence. Stakeholders with a focus on improving equity in diabetes‐related morbidity and mortality should consider adopting VBID strategies such as the ones incorporated into the DHP.

## Supporting information


**Data S1.** Supporting Information.
