# Supplementary material for: The diabetes health plan and medication adherence among individuals with low incomes
Source: Health Serv Res. 2022 May 2;57(Suppl 2):214–21. doi: 10.1111/1475-6773.13992 (PMC9660410; doi:10.1111/1475-6773.13992)
Supplement: Supplementary file 1 — Data S1. Supporting Information. [file HESR-57-214-s001.docx]

| **Supplementary Table 1 Predicted Change in Oral Hypoglycemic Adherence with DHP Exposure, Relative to No Exposure, by Baseline Adherence <$40K (Difference-In-Difference-In-Differences)** | | |
| --- | --- | --- |
| **Enrollment Strategy** | **Opt-In** | |
| **Low Adherence**  **(PDC<80%)** | DHP N = 238  Ctrl N = 350 | +1.7 (-3.4 – 6.8) |
| **High Adherence**  **(PDC ≥80%)** | DHP N = 346  Ctrl N = 542 | +1.3 (-1.7 – 4.3) |
| **Absolute Difference** |  | +0.4 (-5.5 – 6.3) |
| ***p*-value** |  | 0.885 |
| The point estimates reflect percentage point changes in the predicted adherence measure. Ctrl= Matched Control | | |

| **Supplementary Table 2 Predicted Change in Frequency of A1c Testing with DHP Exposure, Relative to No Exposure, by Baseline Adherence (Difference-In-Difference-In-Differences)** | | |
| --- | --- | --- |
| **Enrollment Strategy** | **Opt-In** | |
| **Low Adherence**  **(PDC<80%)** | DHP N = 86  Ctrl N = 122 | -0.44 (-0.96 – 0.09) |
| **High Adherence**  **(PDC ≥80%)** | DHP N = 136  Ctrl N = 197 | -0.23 (-0.56 – 0.11) |
| **Absolute Difference** |  | -0.21 (-0.83 – 0.41) |
| ***p*-value** |  | 0.513 |
| The point estimates reflect change in the number of A1c tests performed from baseline to Year 1. Linear regression models were used to generate effect estimates. | | |
